# 3,6-Dihydr­oxy-2′-[(2-hydr­oxy-1-naphth­yl)methyl­eneamino]xanthene-9-spiro-1′-isoindolin-3′-one acetonitrile solvate

**DOI:** 10.1107/S1600536808000974

**Published:** 2008-01-16

**Authors:** Pei-San Wang, Gen-Hua Wu

**Affiliations:** a Anhui Key Laboratory of Functional Coordination Compounds, School of Chemistry and Chemical Engineering, Anqing Normal College, Anqing 246003, People’s Republic of China; b School of Chemistry and Materials Science, Anhui Normal University, Wuhu 241000, People’s Republic of China

## Abstract

The title compound, C_31_H_20_N_2_O_5_·C_2_H_3_N, was synthesized by the reaction of fluorescein hydrazide and excess 2-hydr­oxy-1-naphthaldehyde in acetonitrile. The spirolactam ring is planar and is nearly at right angles to the two benzene rings of the xanthene system. The dihedral angle between the two benzene rings of the xanthene system is 9.92 (4)°. In the crystal structure, the mol­ecules are linked into extended two-dimensional networks by inter­molecular hydrogen bonding. Acetonitrile mol­ecules are located in the voids between the two-dimensional networks.

## Related literature

For general background, see: Chen *et al.*, (2006 ▶). For related literature, see: Wu *et al.*, (2007 ▶).
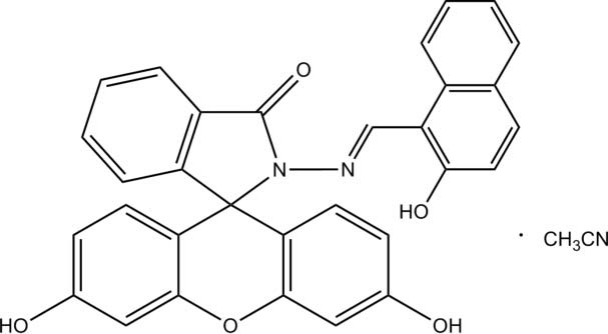


## Experimental

### 

#### Crystal data

C_31_H_20_N_2_O_5_·C_2_H_3_N
*M* *_r_* = 541.54
Monoclinic, 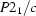

*a* = 18.729 (5) Å
*b* = 15.572 (4) Å
*c* = 9.021 (2) Å
β = 98.495 (4)°
*V* = 2601.9 (11) Å^3^
*Z* = 4
Mo *K*α radiation
μ = 0.10 mm^−1^
*T* = 293 (2) K
0.26 × 0.22 × 0.16 mm

#### Data collection

Bruker SMART APEX CCD area-detector diffractometer
Absorption correction: multi-scan (*SADABS*; Sheldrick, 2000 ▶) *T*_min_ = 0.976, *T*_max_ = 0.985
12963 measured reflections
4627 independent reflections
3272 reflections with *I* > 2σ(*I*)
*R*_int_ = 0.047

#### Refinement

*R*[*F* ^2^ > 2σ(*F* ^2^)] = 0.037
*wR*(*F* ^2^) = 0.098
*S* = 1.03
4627 reflections
375 parameters
H-atom parameters constrained
Δρ_max_ = 0.14 e Å^−3^
Δρ_min_ = −0.15 e Å^−3^

### 

Data collection: *SMART* (Bruker, 1997 ▶); cell refinement: *SAINT-Plus* (Bruker, 1997 ▶); data reduction: *SAINT-Plus*; program(s) used to solve structure: *SHELXS97* (Sheldrick, 2008 ▶); program(s) used to refine structure: *SHELXL97* (Sheldrick, 2008 ▶); molecular graphics: *XP* (Bruker, 2000 ▶); software used to prepare material for publication: *SHELXTL* (Sheldrick, 2008 ▶).

## Supplementary Material

Crystal structure: contains datablocks I, global. DOI: 10.1107/S1600536808000974/at2535sup1.cif

Structure factors: contains datablocks I. DOI: 10.1107/S1600536808000974/at2535Isup2.hkl

Additional supplementary materials:  crystallographic information; 3D view; checkCIF report

## Figures and Tables

**Table 1:** Hydrogen-bond geometry (Å, °)

*D*—H⋯*A*	*D*—H	H⋯*A*	*D*⋯*A*	*D*—H⋯*A*
O4—H4⋯N1	0.82	1.83	2.5600 (16)	147
O3—H6⋯N3^i^	0.82	2.08	2.882 (2)	165
O1—H1⋯O4^ii^	0.82	1.94	2.7484 (16)	170

Symmetry codes: (i) 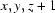; (ii) 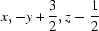.

**Fig. 1.**
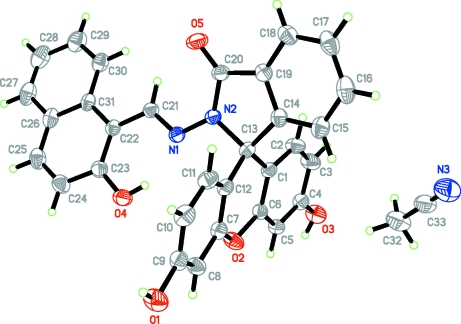
The asymmetric unit, with the atom-numbering scheme. Displacement ellipsoids are drawn at the 30% probability level.

**Fig. 2.**
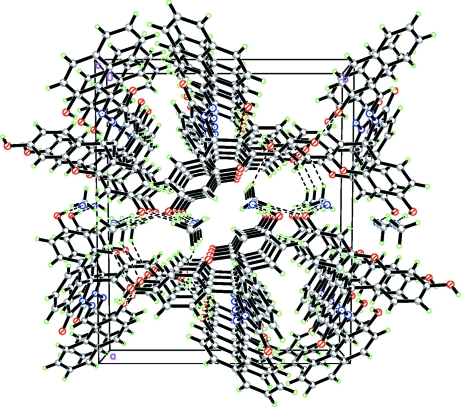
A packing diagram for (I). Hydrogen bonds are shown as dashed lines.

## supplementary crystallographic information

### Comment

Fluorescein dyes have been used extensively for conjugation with biomolecules, owing to their excellent fluorescence properties. A few Fluorescein have also been used as fluorescent chemosensors for metal ions. It was reported that rhodamine B hydrazide could be used as a fluorescent probe for Cu^2+^(Chen *et al.*, 2006). In addition, Fluorescein-based fluorescent chemosensors have received increasing interest in recent years by virtue of their long-wavelength emission and availability. In our previous research using 2-pyridinecarbaldehyde and rhodamine 6 G hydrazide synthesized probe (Wu *et al.*, 2007). The structures are similar with rhodamine 6 G hydrazone probe and fluorescein hydrazone probe. As an extension of our work on this series of complexes, we herein report the crystal structure of the title comound.

The asymmetric unit contains one organic molecule and one acetonitriler molecule. The benzene ring of phenol deviates only slightly from planarity with a dihedral angle of 9.12 (3)°. The water O atom acts as a hydrogen bond acceptor and donor from the hydroxy group in a neighouring organic molecule, thereby forming extended 2-D networks (Table1, Fig. 2). Acetonitrile molecules are located in the voids between the two-dimensional networks.

### Experimental

Briefly, to a suspended solution of fluorescein (300 mg, 0.9 mmol) in CH_3_OH (15 ml), an excess of hydrazine hydrate (1.2 ml, 36 mmol) was added, and the reaction mixture was refluxed for 5 h with stirring. The resulting clear orange solution was evaporated *in vacuo* to give a brown oil, which was then recrystallized from ethanol–water, affording 1 as a light orange crystal (230 mg, yield 70%). Fluorescein hydrazide (0.46 g, 1 mmol) was dissolved in 20 ml absolute acetonitrile. An excessive 2-hydroxy-1-naphthaldehyde (4 mmol) was added then the mixture was refluxed in an air bath for 6 h. After that, the solution was cooled and allowed to stand at room temperature overnight. The yellow single-crystal which appeared after ten days was growed.

### Refinement

All H atoms were positioned geometrically (C—H = 0.93 - 0.96 Å and O—H = 0.82 Å), and refined using a riding model, with *U*_iso_(H) = 1.2 or 1.5*U*_eq_(C or O).

### Crystal data

**Table:**

C_31_H_20_N_2_O_5_·C_2_H_3_N	*F*_000_ = 1128
*M**_r_* = 541.54	*D*_x_ = 1.382 Mg m^−^^3^
Monoclinic, *P*2_1_/*c*	Mo *K*α radiation λ = 0.71073 Å
Hall symbol: -P 2ybc	Cell parameters from 4034 reflections
*a* = 18.729 (5) Å	θ = 2.4–27.0º
*b* = 15.572 (4) Å	µ = 0.10 mm^−^^1^
*c* = 9.021 (2) Å	*T* = 293 (2) K
β = 98.495 (4)º	Block, yellow
*V* = 2601.9 (11) Å^3^	0.26 × 0.22 × 0.16 mm
*Z* = 4	

### Data collection

**Table:**

Bruker SMART APEX CCD area-detector diffractometer	4627 independent reflections
Radiation source: sealed tube	3272 reflections with *I* > 2σ(*I*)
Monochromator: graphite	*R*_int_ = 0.047
*T* = 293(2) K	θ_max_ = 25.1º
φ and ω scans	θ_min_ = 1.7º
Absorption correction: multi-scan(SADABS; Sheldrick, 2000)	*h* = −22→20
*T*_min_ = 0.976, *T*_max_ = 0.985	*k* = −17→18
12963 measured reflections	*l* = −10→10

### Refinement

**Table:**

Refinement on *F*^2^	Hydrogen site location: inferred from neighbouring sites
Least-squares matrix: full	H-atom parameters constrained
*R*[*F*^2^ > 2σ(*F*^2^)] = 0.037	*w* = 1/[σ^2^(*F*_o_^2^) + (0.0455*P*)^2^] where *P* = (*F*_o_^2^ + 2*F*_c_^2^)/3
*wR*(*F*^2^) = 0.098	(Δ/σ)_max_ = 0.001
*S* = 1.03	Δρ_max_ = 0.14 e Å^−^^3^
4627 reflections	Δρ_min_ = −0.15 e Å^−^^3^
375 parameters	Extinction correction: SHELXL97 (Sheldrick, 1997), Fc^*^=kFc[1+0.001xFc^2^λ^3^/sin(2θ)]^-1/4^
Primary atom site location: structure-invariant direct methods	Extinction coefficient: 0.0070 (7)
Secondary atom site location: difference Fourier map	

### Special details

**Table:**

Geometry. All e.s.d.'s (except the e.s.d. in the dihedral angle between two l.s. planes) are estimated using the full covariance matrix. The cell e.s.d.'s are taken into account individually in the estimation of e.s.d.'s in distances, angles and torsion angles; correlations between e.s.d.'s in cell parameters are only used when they are defined by crystal symmetry. An approximate (isotropic) treatment of cell e.s.d.'s is used for estimating e.s.d.'s involving l.s. planes.
Refinement. Refinement of *F*^2^ against ALL reflections. The weighted *R*-factor *wR* and goodness of fit *S* are based on *F*^2^, conventional *R*-factors *R* are based on *F*, with *F* set to zero for negative *F*^2^. The threshold expression of *F*^2^ > σ(*F*^2^) is used only for calculating *R*-factors(gt) *etc*. and is not relevant to the choice of reflections for refinement. *R*-factors based on *F*^2^ are statistically about twice as large as those based on *F*, and *R*- factors based on ALL data will be even larger.

### Fractional atomic coordinates and isotropic or equivalent isotropic displacement parameters (Å^2^)

**Table:**

	*x*	*y*	*z*	*U*_iso_*/*U*_eq_	
C1	0.33564 (7)	0.42174 (9)	0.79315 (17)	0.0395 (4)	
C2	0.35217 (9)	0.33479 (10)	0.7884 (2)	0.0545 (4)	
H2	0.3272	0.3011	0.7128	0.065*	
C3	0.40416 (9)	0.29689 (10)	0.8916 (2)	0.0574 (5)	
H7	0.4137	0.2385	0.8861	0.069*	
C4	0.44199 (8)	0.34666 (10)	1.00373 (18)	0.0457 (4)	
C5	0.42780 (8)	0.43291 (9)	1.01071 (17)	0.0425 (4)	
H5	0.4536	0.4667	1.0851	0.051*	
C6	0.37473 (7)	0.46933 (9)	0.90608 (16)	0.0394 (4)	
C7	0.31973 (8)	0.59927 (9)	0.81305 (17)	0.0419 (4)	
C8	0.32082 (8)	0.68757 (10)	0.82713 (18)	0.0480 (4)	
H8	0.3494	0.7135	0.9078	0.058*	
C9	0.27929 (8)	0.73684 (10)	0.72100 (19)	0.0479 (4)	
C10	0.23642 (8)	0.69775 (10)	0.60184 (19)	0.0533 (4)	
H10	0.2083	0.7308	0.5297	0.064*	
C11	0.23580 (8)	0.60990 (10)	0.59104 (19)	0.0504 (4)	
H11	0.2067	0.5842	0.5108	0.060*	
C12	0.27727 (7)	0.55775 (9)	0.69603 (17)	0.0405 (4)	
C13	0.27427 (7)	0.46102 (9)	0.68603 (16)	0.0396 (4)	
C14	0.26945 (8)	0.42604 (9)	0.52772 (17)	0.0437 (4)	
C15	0.31751 (9)	0.43535 (11)	0.4270 (2)	0.0594 (5)	
H15	0.3601	0.4662	0.4521	0.071*	
C16	0.30051 (11)	0.39730 (12)	0.2870 (2)	0.0693 (5)	
H16	0.3318	0.4039	0.2169	0.083*	
C17	0.23812 (11)	0.34980 (12)	0.2495 (2)	0.0635 (5)	
H17	0.2284	0.3243	0.1555	0.076*	
C18	0.19056 (9)	0.34007 (10)	0.34988 (18)	0.0548 (4)	
H18	0.1486	0.3079	0.3256	0.066*	
C19	0.20660 (8)	0.37939 (9)	0.48858 (17)	0.0436 (4)	
C20	0.16357 (8)	0.38148 (10)	0.61280 (17)	0.0448 (4)	
C21	0.13128 (7)	0.42358 (9)	0.91136 (17)	0.0408 (4)	
H21	0.1084	0.3753	0.8659	0.049*	
C22	0.10663 (7)	0.46010 (9)	1.04201 (16)	0.0396 (4)	
C23	0.13326 (8)	0.53795 (10)	1.10112 (17)	0.0462 (4)	
C24	0.10413 (10)	0.57803 (12)	1.21788 (19)	0.0598 (5)	
H24	0.1220	0.6309	1.2540	0.072*	
C25	0.05010 (10)	0.54010 (13)	1.27822 (19)	0.0622 (5)	
H25	0.0308	0.5679	1.3544	0.075*	
C26	0.02242 (8)	0.45900 (11)	1.22770 (18)	0.0518 (4)	
C27	−0.03305 (9)	0.41847 (14)	1.2928 (2)	0.0682 (5)	
H27	−0.0526	0.4462	1.3689	0.082*	
C28	−0.05798 (11)	0.34026 (15)	1.2463 (2)	0.0771 (6)	
H28	−0.0945	0.3143	1.2900	0.093*	
C29	−0.02859 (9)	0.29812 (13)	1.1316 (2)	0.0708 (5)	
H29	−0.0452	0.2437	1.1010	0.085*	
C30	0.02372 (8)	0.33577 (11)	1.06483 (19)	0.0548 (4)	
H30	0.0421	0.3069	0.9884	0.066*	
C31	0.05084 (7)	0.41780 (10)	1.10902 (17)	0.0431 (4)	
C32	0.54315 (12)	0.38827 (12)	0.6728 (2)	0.0826 (6)	
H32A	0.5930	0.3955	0.7141	0.124*	
H32B	0.5265	0.3336	0.7028	0.124*	
H32C	0.5151	0.4332	0.7087	0.124*	
C33	0.53548 (10)	0.39211 (12)	0.5109 (3)	0.0678 (5)	
N1	0.18474 (6)	0.45736 (8)	0.85739 (13)	0.0403 (3)	
N2	0.20397 (6)	0.42793 (7)	0.72513 (13)	0.0404 (3)	
N3	0.52922 (10)	0.39621 (12)	0.3840 (2)	0.0900 (6)	
O1	0.28347 (6)	0.82387 (7)	0.73845 (14)	0.0670 (4)	
H1	0.2542	0.8470	0.6743	0.100*	
O2	0.36425 (6)	0.55596 (6)	0.92433 (12)	0.0537 (3)	
O3	0.49339 (6)	0.30663 (7)	1.10332 (14)	0.0644 (4)	
H6	0.5067	0.3389	1.1738	0.097*	
O4	0.18721 (6)	0.58052 (7)	1.04607 (13)	0.0589 (3)	
H4	0.1992	0.5533	0.9759	0.088*	
O5	0.10443 (6)	0.35036 (8)	0.61789 (13)	0.0678 (4)	

### Atomic displacement parameters (Å^2^)

**Table:**

	*U*^11^	*U*^22^	*U*^33^	*U*^12^	*U*^13^	*U*^23^
C1	0.0349 (8)	0.0383 (9)	0.0458 (9)	−0.0024 (6)	0.0074 (7)	0.0024 (7)
C2	0.0552 (10)	0.0407 (10)	0.0634 (11)	−0.0034 (8)	−0.0047 (9)	−0.0041 (8)
C3	0.0630 (11)	0.0349 (9)	0.0695 (12)	0.0048 (8)	−0.0057 (9)	−0.0008 (8)
C4	0.0447 (9)	0.0421 (9)	0.0497 (10)	0.0035 (7)	0.0053 (8)	0.0050 (7)
C5	0.0452 (9)	0.0405 (9)	0.0407 (9)	−0.0021 (7)	0.0024 (7)	−0.0003 (7)
C6	0.0427 (9)	0.0329 (8)	0.0436 (9)	0.0006 (6)	0.0099 (7)	0.0016 (7)
C7	0.0416 (9)	0.0408 (9)	0.0432 (9)	0.0053 (7)	0.0059 (7)	0.0061 (7)
C8	0.0501 (9)	0.0400 (9)	0.0524 (10)	0.0037 (7)	0.0025 (8)	−0.0007 (7)
C9	0.0491 (10)	0.0381 (9)	0.0575 (11)	0.0055 (7)	0.0115 (8)	0.0073 (8)
C10	0.0489 (10)	0.0502 (11)	0.0593 (11)	0.0057 (8)	0.0032 (9)	0.0163 (8)
C11	0.0435 (9)	0.0520 (11)	0.0534 (11)	−0.0026 (7)	−0.0004 (8)	0.0077 (8)
C12	0.0353 (8)	0.0411 (9)	0.0459 (9)	−0.0010 (6)	0.0090 (7)	0.0047 (7)
C13	0.0345 (8)	0.0429 (9)	0.0419 (9)	−0.0031 (6)	0.0077 (7)	0.0020 (7)
C14	0.0431 (9)	0.0453 (9)	0.0428 (9)	0.0058 (7)	0.0067 (7)	0.0032 (7)
C15	0.0573 (10)	0.0682 (12)	0.0561 (12)	0.0001 (9)	0.0196 (9)	0.0022 (9)
C16	0.0804 (14)	0.0795 (14)	0.0530 (12)	0.0217 (11)	0.0267 (11)	0.0086 (10)
C17	0.0785 (13)	0.0681 (12)	0.0422 (11)	0.0274 (10)	0.0039 (10)	−0.0020 (9)
C18	0.0604 (11)	0.0537 (11)	0.0466 (10)	0.0122 (8)	−0.0037 (9)	−0.0050 (8)
C19	0.0459 (9)	0.0440 (9)	0.0396 (9)	0.0055 (7)	0.0020 (7)	0.0014 (7)
C20	0.0419 (9)	0.0451 (9)	0.0459 (10)	−0.0040 (7)	0.0017 (7)	−0.0033 (7)
C21	0.0386 (8)	0.0379 (8)	0.0457 (9)	−0.0019 (7)	0.0052 (7)	0.0007 (7)
C22	0.0364 (8)	0.0437 (9)	0.0376 (9)	0.0050 (7)	0.0018 (7)	0.0039 (7)
C23	0.0454 (9)	0.0509 (10)	0.0413 (9)	0.0020 (7)	0.0028 (7)	−0.0011 (7)
C24	0.0641 (11)	0.0649 (12)	0.0489 (11)	0.0051 (9)	0.0039 (9)	−0.0121 (9)
C25	0.0634 (11)	0.0820 (14)	0.0417 (10)	0.0176 (10)	0.0092 (9)	−0.0053 (9)
C26	0.0462 (9)	0.0688 (12)	0.0410 (10)	0.0137 (8)	0.0081 (8)	0.0133 (8)
C27	0.0582 (11)	0.0988 (16)	0.0508 (11)	0.0168 (11)	0.0188 (9)	0.0190 (11)
C28	0.0636 (12)	0.0927 (17)	0.0803 (15)	−0.0013 (11)	0.0278 (11)	0.0310 (12)
C29	0.0632 (12)	0.0694 (13)	0.0838 (14)	−0.0063 (10)	0.0240 (11)	0.0190 (11)
C30	0.0503 (10)	0.0556 (11)	0.0611 (11)	0.0010 (8)	0.0166 (9)	0.0118 (9)
C31	0.0378 (8)	0.0511 (10)	0.0398 (9)	0.0096 (7)	0.0040 (7)	0.0103 (7)
C32	0.1057 (17)	0.0644 (13)	0.0762 (15)	−0.0114 (11)	0.0088 (13)	−0.0104 (11)
C33	0.0679 (13)	0.0562 (12)	0.0775 (16)	−0.0092 (9)	0.0048 (12)	−0.0128 (11)
N1	0.0388 (7)	0.0432 (7)	0.0389 (7)	−0.0020 (5)	0.0059 (6)	−0.0012 (6)
N2	0.0366 (7)	0.0449 (7)	0.0399 (7)	−0.0070 (5)	0.0068 (6)	−0.0054 (6)
N3	0.0966 (14)	0.0927 (14)	0.0773 (13)	−0.0082 (10)	0.0014 (12)	−0.0090 (11)
O1	0.0785 (9)	0.0400 (7)	0.0783 (9)	0.0095 (6)	−0.0022 (7)	0.0083 (6)
O2	0.0680 (7)	0.0367 (6)	0.0509 (7)	0.0085 (5)	−0.0094 (6)	−0.0027 (5)
O3	0.0701 (8)	0.0492 (7)	0.0668 (8)	0.0141 (6)	−0.0135 (7)	0.0053 (6)
O4	0.0616 (7)	0.0571 (7)	0.0596 (8)	−0.0153 (6)	0.0145 (6)	−0.0149 (6)
O5	0.0510 (7)	0.0878 (9)	0.0651 (8)	−0.0290 (6)	0.0100 (6)	−0.0191 (7)

### Geometric parameters (Å, °)

**Table:**

C1—C6	1.379 (2)	C18—H18	0.9300
C1—C2	1.391 (2)	C19—C20	1.474 (2)
C1—C13	1.5168 (19)	C20—O5	1.2161 (17)
C2—C3	1.377 (2)	C20—N2	1.3769 (18)
C2—H2	0.9300	C21—N1	1.2885 (17)
C3—C4	1.383 (2)	C21—C22	1.444 (2)
C3—H7	0.9300	C21—H21	0.9300
C4—C5	1.372 (2)	C22—C23	1.387 (2)
C4—O3	1.3663 (18)	C22—C31	1.441 (2)
C5—C6	1.3867 (19)	C23—O4	1.3620 (18)
C5—H5	0.9300	C23—C24	1.402 (2)
C6—O2	1.3767 (17)	C24—C25	1.353 (2)
C7—C8	1.381 (2)	C24—H24	0.9300
C7—O2	1.3821 (17)	C25—C26	1.415 (2)
C7—C12	1.384 (2)	C25—H25	0.9300
C8—C9	1.375 (2)	C26—C31	1.418 (2)
C8—H8	0.9300	C26—C27	1.415 (2)
C9—O1	1.3652 (18)	C27—C28	1.349 (3)
C9—C10	1.384 (2)	C27—H27	0.9300
C10—C11	1.371 (2)	C28—C29	1.404 (3)
C10—H10	0.9300	C28—H28	0.9300
C11—C12	1.394 (2)	C29—C30	1.357 (2)
C11—H11	0.9300	C29—H29	0.9300
C12—C13	1.510 (2)	C30—C31	1.410 (2)
C13—N2	1.5036 (17)	C30—H30	0.9300
C13—C14	1.519 (2)	C32—C33	1.447 (3)
C14—C15	1.378 (2)	C32—H32A	0.9600
C14—C19	1.384 (2)	C32—H32B	0.9600
C15—C16	1.389 (3)	C32—H32C	0.9600
C15—H15	0.9300	C33—N3	1.135 (2)
C16—C17	1.382 (3)	N1—N2	1.3747 (16)
C16—H16	0.9300	O1—H1	0.8200
C17—C18	1.369 (2)	O3—H6	0.8200
C17—H17	0.9300	O4—H4	0.8200
C18—C19	1.385 (2)		
			
C6—C1—C2	116.72 (13)	C19—C18—H18	120.9
C6—C1—C13	121.57 (13)	C18—C19—C14	121.68 (15)
C2—C1—C13	121.59 (13)	C18—C19—C20	128.78 (15)
C3—C2—C1	122.31 (15)	C14—C19—C20	109.53 (13)
C3—C2—H2	118.8	O5—C20—N2	125.88 (14)
C1—C2—H2	118.8	O5—C20—C19	128.55 (14)
C2—C3—C4	119.27 (15)	N2—C20—C19	105.57 (13)
C2—C3—H7	120.4	N1—C21—C22	120.49 (14)
C4—C3—H7	120.4	N1—C21—H21	119.8
C5—C4—O3	122.60 (14)	C22—C21—H21	119.8
C5—C4—C3	120.01 (14)	C23—C22—C31	118.68 (14)
O3—C4—C3	117.39 (14)	C23—C22—C21	121.17 (14)
C4—C5—C6	119.55 (14)	C31—C22—C21	120.08 (14)
C4—C5—H5	120.2	O4—C23—C22	121.95 (14)
C6—C5—H5	120.2	O4—C23—C24	116.69 (15)
O2—C6—C1	123.04 (13)	C22—C23—C24	121.33 (15)
O2—C6—C5	114.82 (13)	C25—C24—C23	120.32 (17)
C1—C6—C5	122.14 (14)	C25—C24—H24	119.8
C8—C7—O2	114.83 (13)	C23—C24—H24	119.8
C8—C7—C12	122.28 (14)	C24—C25—C26	121.34 (16)
O2—C7—C12	122.88 (14)	C24—C25—H25	119.3
C9—C8—C7	119.51 (15)	C26—C25—H25	119.3
C9—C8—H8	120.2	C31—C26—C27	119.49 (17)
C7—C8—H8	120.2	C31—C26—C25	119.12 (15)
O1—C9—C8	117.18 (15)	C27—C26—C25	121.39 (17)
O1—C9—C10	122.88 (14)	C28—C27—C26	120.99 (18)
C8—C9—C10	119.93 (15)	C28—C27—H27	119.5
C11—C10—C9	119.46 (15)	C26—C27—H27	119.5
C11—C10—H10	120.3	C27—C28—C29	119.73 (17)
C9—C10—H10	120.3	C27—C28—H28	120.1
C10—C11—C12	122.34 (15)	C29—C28—H28	120.1
C10—C11—H11	118.8	C30—C29—C28	120.82 (19)
C12—C11—H11	118.8	C30—C29—H29	119.6
C7—C12—C11	116.47 (14)	C28—C29—H29	119.6
C7—C12—C13	121.57 (13)	C29—C30—C31	121.30 (17)
C11—C12—C13	121.93 (13)	C29—C30—H30	119.3
N2—C13—C12	110.73 (11)	C31—C30—H30	119.3
N2—C13—C1	108.65 (11)	C26—C31—C30	117.62 (14)
C12—C13—C1	110.26 (12)	C26—C31—C22	119.09 (15)
N2—C13—C14	99.51 (11)	C30—C31—C22	123.27 (14)
C12—C13—C14	114.24 (12)	C33—C32—H32A	109.5
C1—C13—C14	112.87 (12)	C33—C32—H32B	109.5
C15—C14—C19	120.07 (15)	H32A—C32—H32B	109.5
C15—C14—C13	128.93 (14)	C33—C32—H32C	109.5
C19—C14—C13	111.00 (13)	H32A—C32—H32C	109.5
C14—C15—C16	118.06 (17)	H32B—C32—H32C	109.5
C14—C15—H15	121.0	N3—C33—C32	179.1 (2)
C16—C15—H15	121.0	C21—N1—N2	120.63 (12)
C17—C16—C15	121.52 (17)	N1—N2—C20	128.89 (12)
C17—C16—H16	119.2	N1—N2—C13	116.11 (11)
C15—C16—H16	119.2	C20—N2—C13	114.35 (12)
C18—C17—C16	120.44 (17)	C9—O1—H1	109.5
C18—C17—H17	119.8	C6—O2—C7	118.21 (11)
C16—C17—H17	119.8	C4—O3—H6	109.5
C17—C18—C19	118.22 (17)	C23—O4—H4	109.5
C17—C18—H18	120.9		
			
C6—C1—C2—C3	0.8 (2)	C15—C14—C19—C18	0.9 (2)
C13—C1—C2—C3	−175.13 (15)	C13—C14—C19—C18	−179.13 (13)
C1—C2—C3—C4	−0.6 (3)	C15—C14—C19—C20	−178.10 (14)
C2—C3—C4—C5	−0.3 (2)	C13—C14—C19—C20	1.88 (17)
C2—C3—C4—O3	−179.62 (15)	C18—C19—C20—O5	−1.5 (3)
O3—C4—C5—C6	−179.86 (14)	C14—C19—C20—O5	177.43 (16)
C3—C4—C5—C6	0.9 (2)	C18—C19—C20—N2	179.33 (15)
C2—C1—C6—O2	179.93 (13)	C14—C19—C20—N2	−1.77 (17)
C13—C1—C6—O2	−4.1 (2)	N1—C21—C22—C23	8.3 (2)
C2—C1—C6—C5	−0.3 (2)	N1—C21—C22—C31	−174.81 (12)
C13—C1—C6—C5	175.71 (13)	C31—C22—C23—O4	178.31 (13)
C4—C5—C6—O2	179.24 (13)	C21—C22—C23—O4	−4.7 (2)
C4—C5—C6—C1	−0.6 (2)	C31—C22—C23—C24	−3.8 (2)
O2—C7—C8—C9	179.00 (13)	C21—C22—C23—C24	173.18 (13)
C12—C7—C8—C9	−0.9 (2)	O4—C23—C24—C25	179.68 (15)
C7—C8—C9—O1	−178.66 (14)	C22—C23—C24—C25	1.6 (3)
C7—C8—C9—C10	0.5 (2)	C23—C24—C25—C26	1.1 (3)
O1—C9—C10—C11	179.17 (15)	C24—C25—C26—C31	−1.5 (2)
C8—C9—C10—C11	0.1 (2)	C24—C25—C26—C27	178.67 (16)
C9—C10—C11—C12	−0.3 (2)	C31—C26—C27—C28	1.7 (3)
C8—C7—C12—C11	0.7 (2)	C25—C26—C27—C28	−178.46 (17)
O2—C7—C12—C11	−179.20 (13)	C26—C27—C28—C29	0.0 (3)
C8—C7—C12—C13	−177.30 (14)	C27—C28—C29—C30	−1.3 (3)
O2—C7—C12—C13	2.8 (2)	C28—C29—C30—C31	0.7 (3)
C10—C11—C12—C7	−0.1 (2)	C27—C26—C31—C30	−2.2 (2)
C10—C11—C12—C13	177.87 (14)	C25—C26—C31—C30	177.93 (14)
C7—C12—C13—N2	106.04 (15)	C27—C26—C31—C22	179.15 (13)
C11—C12—C13—N2	−71.84 (17)	C25—C26—C31—C22	−0.7 (2)
C7—C12—C13—C1	−14.24 (18)	C29—C30—C31—C26	1.1 (2)
C11—C12—C13—C1	167.88 (13)	C29—C30—C31—C22	179.63 (15)
C7—C12—C13—C14	−142.61 (14)	C23—C22—C31—C26	3.2 (2)
C11—C12—C13—C14	39.52 (19)	C21—C22—C31—C26	−173.75 (13)
C6—C1—C13—N2	−106.63 (15)	C23—C22—C31—C30	−175.28 (14)
C2—C1—C13—N2	69.15 (17)	C21—C22—C31—C30	7.7 (2)
C6—C1—C13—C12	14.90 (18)	C22—C21—N1—N2	−173.76 (12)
C2—C1—C13—C12	−169.33 (13)	C21—N1—N2—C20	19.9 (2)
C6—C1—C13—C14	144.01 (14)	C21—N1—N2—C13	−169.86 (12)
C2—C1—C13—C14	−40.22 (19)	O5—C20—N2—N1	−7.9 (3)
N2—C13—C14—C15	178.80 (15)	C19—C20—N2—N1	171.36 (13)
C12—C13—C14—C15	60.8 (2)	O5—C20—N2—C13	−178.20 (15)
C1—C13—C14—C15	−66.2 (2)	C19—C20—N2—C13	1.02 (16)
N2—C13—C14—C19	−1.18 (15)	C12—C13—N2—N1	−51.04 (16)
C12—C13—C14—C19	−119.14 (14)	C1—C13—N2—N1	70.20 (15)
C1—C13—C14—C19	113.83 (14)	C14—C13—N2—N1	−171.60 (11)
C19—C14—C15—C16	0.4 (2)	C12—C13—N2—C20	120.60 (14)
C13—C14—C15—C16	−179.55 (15)	C1—C13—N2—C20	−118.17 (14)
C14—C15—C16—C17	−1.3 (3)	C14—C13—N2—C20	0.03 (15)
C15—C16—C17—C18	1.0 (3)	C1—C6—O2—C7	−9.1 (2)
C16—C17—C18—C19	0.3 (2)	C5—C6—O2—C7	171.10 (12)
C17—C18—C19—C14	−1.3 (2)	C8—C7—O2—C6	−170.15 (13)
C17—C18—C19—C20	177.50 (15)	C12—C7—O2—C6	9.8 (2)

### Hydrogen-bond geometry (Å, °)

**Table:**

*D*—H···*A*	*D*—H	H···*A*	*D*···*A*	*D*—H···*A*
O4—H4···N1	0.82	1.83	2.5600 (16)	147
O3—H6···N3^i^	0.82	2.08	2.882 (2)	165
O1—H1···O4^ii^	0.82	1.94	2.7484 (16)	170

Symmetry codes: (i) *x*, *y*, *z*+1; (ii) *x*, −*y*+3/2, *z*−1/2.
